# A European consensus recommendation on the management of delayed methotrexate elimination: supportive measures, leucovorin rescue and glucarpidase treatment

**DOI:** 10.1007/s00432-024-05945-6

**Published:** 2024-10-02

**Authors:** Stefan S. Bielack, Carole Soussain, Christopher P. Fox, Caroline Houillier, Thais Murciano, Wendy Osborne, Pier Luigi Zinzani, Carmelo Rizzari, Stefan Schwartz

**Affiliations:** 1 Paediatrics 5 (Oncology, Haematology, Immunology), Klinikum Stuttgart - Olgahospital, Stuttgart Cancer Centre, Stuttgart, Germany; 2 Service d’Hématologie, Institut Curie, Paris, France; 3 School of Medicine, University of Nottingham, Nottingham, UK; 4 Department of Neurooncology, IHU, ICM, Groupe Hospitalier Pitié-Salpêtrière, AP-HP, Sorbonne Université, Paris, France; 5 Pediatric Oncology and Hematology Service, Vall d’Hebron Hospital Universitari, Vall d’Hebron Barcelona Hospital Campus, Barcelona, Spain; 6 Department of Haematology, Newcastle Upon Tyne NHS Foundation Trust, and Newcastle University, Newcastle Upon Tyne, UK; 7 Istituto di Ematologia “Seràgnoli”, IRCCS Azienda Ospedaliero-Universitaria di Bologna, Bologna, Italy; 8 Dipartimento di Scienze Mediche e Chirurgiche, Università di Bologna, Bologna, Italy; 9 Department of Pediatrics, Foundation IRCCS San Gerardo dei Tintori, Monza, Italy; 10 Department of Medicine and Surgery, University of Milano-Bicocca, Milan, Italy; 11 Department of Hematology, Oncology and Tumor Immunology, Charité-Universitätsmedizin Berlin, Corporate Member of Freie Universität and Humboldt-Universität zu Berlin, Campus Benjamin Franklin, Berlin, Germany

**Keywords:** Consensus, Methotrexate pharmacokinetics, Carboxypeptidases, Delayed methotrexate elimination, Glucarpidase, Methotrexate toxicity

## Abstract

High-dose methotrexate (HDMTX) is used in the treatment of a range of adult and childhood cancers. Although HDMTX can provide effective anti-tumor activity with an acceptable safety profile for most patients, delayed methotrexate elimination (DME) develops in a minority of patients receiving HDMTX and may be accompanied by renal dysfunction and potentially life-threatening toxicity. A panel of European physicians with experience in the use of HDMTX as well as of glucarpidase convened to develop a series of consensus statements to provide practical guidance on the prevention and treatment of DME, including the use of glucarpidase. Robust implementation of supportive measures including hyperhydration and urine alkalinization emerged as critical in order to reduce the risk of DME with HDMTX treatment, with leucovorin rescue critical in reducing the risk of DME complications. Early recognition of DME is important to promptly implement appropriate treatment including, intensified hydration, high-dose leucovorin and, when appropriate, glucarpidase.

## Introduction

Methotrexate (MTX) is an antifolate agent used in the treatment of various types of cancer as well as autoimmune disorders including rheumatoid arthritis, psoriasis and Crohn’s disease (Widemann and Adamson 2006; Howard et al. 2016). MTX dosing regimens vary widely, depending on the indication. In non-oncological settings, oral doses of 2.5‒30 mg weekly are typical, and oral MTX at doses of 20–40 mg/m^2^/week is also used alongside 6-mercaptopurine as maintenance therapy for acute lymphoblastic leukaemia (ALL) (Toksvang et al. 2022). High-dose MTX (HDMTX) therapy is generally defined as a dose of ≥ 500 mg/m^2^ administered by intravenous infusion and has a number of oncological indications; these include treatment of ALL, lymphomas and osteosarcoma, as well as prophylaxis for selected patients with lymphoma considered to be at high risk of central nervous system (CNS) involvement, while cranial irradiation may be replaced by HDMTX in patients with lymphoma at low risk for CNS involvement (medac GmbH 2022; Isakoff et al. 2015; Mantadakis et al. 2005; Schaff and Grommes 2022; Fox et al. 2019; Woessmann et al. 2005).

Care is needed when administering HDMTX due to the risk of significant and potentially life-threatening toxicities. During treatment with HDMTX, crystallization of MTX and its metabolites within renal tubules can result in acute kidney injury (AKI) (Garneau et al. 2015). Definitions of HDMTX-associated AKI can vary, but a ≥ 1.5-fold increase in serum creatinine within 4 days of HDMTX is typical (Gupta et al. 2023). As MTX is primarily eliminated via the kidneys, AKI can lead to delayed MTX elimination (DME) and prolonged exposure to toxic levels of MTX, and thereby increased risks of renal, hepatic, haematologic and neurologic toxicities (Howard et al. 2016). Supportive measures including hyperhydration and urine alkalization are critical in reducing the risk of DME, while use of leucovorin rescue is critical in reducing the risk of DME complications (Widemann and Adamson 2006; Howard et al. 2016; Alsdorf et al. 2021). It is important that measures designed to mitigate against the risk of toxicity do not inadvertently impact the pharmacokinetics of MTX such that anti-tumor activity is compromised.

As it is not possible to avoid completely the risk of DME, when it occurs, it requires prompt and effective intervention. Glucarpidase (carboxypeptidase G2) is indicated to reduce toxic plasma MTX levels in adults and children who either have DME or are at risk of MTX toxicity and acts by converting MTX into its inactive metabolites glutamate and 2,4-diamino-N10-methylpteroic acid (DAMPA), which is non-toxic and is excreted in the urine or further metabolized in the liver (SERB SAS 2024; Ramsey et al. 2018). A single dose of glucarpidase results in rapid and substantial reductions in plasma MTX levels, with a > 95% reduction achieved within 15 min of administration (SERB SAS 2024; Schwartz et al. 2007; Widemann et al. 2014).

Consensus guidelines for use of glucarpidase in the clinical management of DME were published in 2018 (Ramsey et al. 2018). However, glucarpidase was not approved in Europe until 2022 (European Medicines Agency 2022). Given differences in the definitions for its use in the European Summary of Product Characteristics and the previously available United States Product Information (BTG International Inc. 2019) for glucarpidase together with evolving protocols in response to new evidence, we sought to develop consensus recommendations on the management of DME and its treatment with glucarpidase in Europe to provide guidance for healthcare providers practicing in this region. With the wider availability of glucarpidase, there is a need for a Europe-specific guideline to inform clinically rational use of glucarpidase, supporting healthcare providers with relevant decision making.

As a group, we have considerable experience in the use of high-dose methotrexate delivery, toxicities and use of glucarpidase. With DME representing a potentially life-threatening emergency, our aim is to provide clear and practical guidance that can be applied in a timely manner. Our experience covers the range of indications where HDMTX is used, encompassing both adult and pediatric settings, thereby taking in different dosing and toxicity profiles in these patient populations.

## Consensus process

As a group of European physicians with experience in the use of HDMTX in the treatment of cancers and glucarpidase for the treatment of DME, we sought to develop a series of consensus statements as a guide to management in clinical practice in Europe. Of 12 individuals invited to participate, nine accepted the invitation. For the three individuals who declined to take part, the primary reason was lack of capacity due to other commitments.

We used a variation of the Delphi method (estimate-talk-estimate) in which participants received a series of iterations of the draft consensus statements for review. Comments and scoring were not anonymized so that input from different specialities could be identified. Participants were also allowed to interact between iterations.

Participants were initially sent a set of open questions from which an initial set of consensus statements was developed, complemented by reference to the literature where appropriate. The initial draft was sent to participants as an online questionnaire. Participants provided quantitative feedback on each statement, rating their agreement with each statement on a scale of 1 (strongly disagree) to 10 (strongly agree). Participants also had the opportunity to provide qualitative input by adding their comments.

The feedback was incorporated into a revised set of statements which were discussed in a videoconference. Participants who were unable to attend the videoconference had the opportunity to provide their input prior to the meeting. A revised set of statements were then circulated by email for further comment and approval.

### Background information

HDMTX is usually defined as a dose of ≥ 500 mg/m^2^ MTX administered by IV infusion. In ALL, HDMTX is typically administered as short (~ 3 h) or long (24–36 h) duration infusions of 1–5 g/m^2^ while short (2–4 h) infusions of at least 3 g/m^2^ per cycle are recommended for CNS lymphoma (Howard et al. 2016; Fox et al. 2019). Higher doses (8–12 g/m^2^) infused over 4 h are used in the treatment of osteosarcoma (Fox et al. 2021; Marina et al. 2016). A dose of ≥ 3 g/m^2^ MTX given as a short duration infusion may be used for prophylaxis of CNS involvement in patients with systemic lymphoma considered to be at high risk for CNS recurrence (Peñalver et al. 2017; McKay et al. 2020). (Table 1; **Statement #1.1**)

**Table 1:** Consensus statements on the management of delayed methotrexate elimination with glucarpidase

**1**	**Background information**
1.1	A variety of HDMTX regimens are in use according to indication and local practice, with MTX doses of 1–12 g/m² infused over periods ranging from a few hours to 36 h.
1.2	DME occurs in approximately 15% of treatment cycles* in patients receiving treatment with high-dose MTX.
1.3	DME may be accompanied by renal dysfunction and is potentially toxic.
1.4	Systemic toxicities can develop in a proportion of patients with DME, with the risk varying according to MTX infusion schedule. Renal and liver toxicity occur more frequently with shorter infusions at higher doses while mucosal and hematological toxicities are more common with longer infusions at lower doses.
1.5	Various definitions exist but DME is typically defined as serum MTX ≥ 10 µmol/L at 24 h (for short MTX infusions), ≥ 1 µmol/L at 42 or 48 h, or ≥ 0.3 µmol/L at 72 h.
1.6	Serum creatinine increases within 24–36 h of HDMTX initiation may be an early indicator of delayed MTX elimination.
**2**	**Risk factors for delayed methotrexate elimination**
2.1	MTX is eliminated predominantly (> 90%) via the kidneys, and patients with a history of renal dysfunction are at increased risk of DME.
2.2	Other clinical risk factors for DME include frailty, excess body weight, presence of pleural effusion and ascites, sepsis, fever/infection, tumor lysis, diabetes or hypoalbuminemia, Down syndrome, and concomitant use of drugs that are nephrotoxic or interfere with MTX elimination.
2.3	Renal impairment should be considered alongside other factors, particularly serum MTX levels, when assessing the risk of MTX toxicity. Risk of MTX toxicity may be increased with mild or more severe impairment (creatinine clearance ≤ 60 mL/min).
2.4	Age should be considered alongside other risk factors when deciding whether to administer HDMTX.
2.5	Acetylsalicylic acid/non-steroidal anti-inflammatory drugs and some antibiotics can increase the risk of DME and should be avoided when receiving MTX. Proton pump inhibitors, tyrosine kinase inhibitors and certain other drugs, as well as cola drinks and fruit juices, can theoretically delay MTX elimination and use or consumption of these should be moderate or avoided. Use of loop diuretics is recommended in exceptional circumstances (as detailed in Statement #3.5).
2.6	In some indications, the dose of MTX may be reduced if risk factors for delayed MTX elimination are present. Dose reduction should be based on a holistic assessment of risk factors and disease state, but with particular consideration to renal impairment.
2.7	When determining the dose of HDMTX for normal and overweight patients, the actual body weight can be used when calculating body surface area; however, this is not appropriate for severely obese patients for whom a dose cap (calculated using the ideal body weight) may be considered.
2.8	Further research is needed to identify and characterize predictors of MTX toxicity and guide decisions on eligibility, dosing and potential early use of glucarpidase.
**3**	**Supportive care**
3.1	Hyperhydration (≥ 2.5 L/m^2^/24 h) with dextrose/saline supplemented with sodium bicarbonate should start several hours before the administration of HDMTX and continue until MTX clearance to non-toxic levels.
3.2	The amount of sodium bicarbonate in the hydration fluid should be adjusted to achieve a urine pH of ≥ 7.
3.3	The urine pH must be ≥ 7 before administering HDMTX.
3.4	HDMTX should not generally be started in patients with signs of infection, fever or vomiting.
3.5	Use of loop diuretics or acetazolamide to maintain diuresis and avoid fluid overload should be used for patients with weight gain/fluid retention and selected patients with severe renal impairment.
3.6	Various protocols for leucovorin rescue of DME are available. Rescue is typically started at 24–36 h after MTX administration with leucovorin given every 6 h at a dose adjusted according to serum MTX levels.
**4**	**Monitoring**
4.1	Most clinics use immunoassay methods to measure serum MTX levels. It is important to note that immunoassays do not reliably distinguish between MTX and its metabolites and are subject to interference following glucarpidase treatment.
4.2	Serum MTX levels should be determined at regular intervals starting 24 h after administration of HDMTX (e.g. 24, 42, 48 and 72 h), and then at least every 24 h until the patient meets discharge criteria.
4.3	Creatinine and/or GFR should be regularly monitored, typically every 24 h starting 24 h after the administration of HDMTX; closer monitoring, including cystatin C where available, is required if DME is suspected.
4.4	Patients should be closely monitored including regular assessment of clinical signs, fluid balance, weight, urine output and urine pH, and renal function.
4.5	Patient discharge can be considered either: • When serum MTX is < 0.1 µmol/L, renal function and electrolytes are stable with no significant fluid overload, and patients are clinically well. Or: • On Day 3 after HDMTX infusion if MTX kinetics at 48 h are favorable (serum MTX < 1 µmol/L) and creatinine stable.
**5**	**Use of glucarpidase**
5.1	The decision whether to administer glucarpidase should be based on plasma MTX levels and should take into account factors including renal function, clinical signs and evidence and/or risk of MTX toxicity.
5.2	Glucarpidase may be considered, especially in the context of impaired renal function, when plasma MTX concentrations are two standard deviations above the mean expected MTX plasma concentration (e.g., as determined via the website MTXPK.org), or if the 24-hour plasma MTX level is above 50 μmol/L, 36-hour level is above 30 µmol/L, 42-hour level is above 10 µmol/L, or 48-hour level is above 5 µmol/L.
5.3	For patients with DME or high risk of MTX toxicity the recommended dose of glucarpidase is 50 units/kg administered by intravenous injection over at least 5 min.
5.4	The decision whether to use glucarpidase should be made at the earliest opportunity and ideally within 48–60 h of MTX infusion start.
5.5	Glucarpidase should be ordered immediately when needed. If stocks of glucarpidase are not locally available, arrangements should be in place to allow access within 24 h and ideally in < 12 h.
5.6	MTX levels should continue to be monitored after administration of glucarpidase, ideally with an HPLC-based assay, until MTX is undetectable. Potential rebound of MTX levels can occur from ~ 48 h after glucarpidase administration; however, the rebound level is typically substantially less than that prior to glucarpidase administration and unlikely to be clinically relevant.
5.7	Leucovorin should be stopped at least 2 h prior to, and restarted at least 2 h after, glucarpidase infusion. Leucovorin should then be continued until serum MTX levels are undetectable.
**6**	**Other strategies for treating delayed methotrexate elimination**
6.1	Intensification of hydration and leucovorin can be used as an alternative to glucarpidase for the treatment of DME.
6.2	If available, dialysis using a high-flux dialyser may be considered on a case-by-case basis for selected patients (e.g. those with severe renal failure and anuria) following consultation with a nephrologist.
6.3	There is insufficient evidence to support the use of activated charcoal or binding agents such as cholestyramine in the treatment of toxicity due to DME.

*DME defined as serum MTX ≥ 0.2 µmol/L at 72 h

DME, delayed methotrexate elimination; GFR, glomerular filtration rate; HDMTX, high-dose methotrexate; HPLC, high-performance liquid chromatography; MTX, methotrexate

The risk of DME associated with HDMTX is influenced by a range of factors. Various definitions of DME have been used, contributing to variability in reported incidence. In a group of patients receiving HDMTX for ALL, aggressive lymphoma or osteosarcoma and based on a definition of DME of serum MTX ≥ 0.2 µmol/L at 72 h, DME was found to occur in approximately 15% of treatment cycles (Alsdorf et al. 2021). DME occurrence may not always be reported appropriately, and so real-world data may underestimate the true incidence of DME. Higher rates of DME have been reported with HDMTX in patients with lymphoma compared with osteosarcoma (May et al. 2014), and with higher doses of MTX in pediatric patients with ALL or lymphoma (Nakano et al. 2021). (**Statement #1.2**)

DME may be accompanied by renal dysfunction and the prolonged exposure to MTX could increase drug toxicity. The median incidence of renal toxicity in 20 trials of HDMTX for osteosarcoma was 1.5% (range: 0.0‒12.4%). Among the 3,887 predominantly adult patients for whom renal toxicity data were available, 23 (0.6%) developed Grade 3/4 nephrotoxicity and three (0.08%) deaths were attributable to HDMTX-induced renal dysfunction (Widemann et al. 2004). A recent study in children with ALL found that nephrotoxicity developed during 1.5‒2.9% of 136 HDMTX cycles (Khera et al. 2023). Two studies evaluating large series of patients with diffuse large B-cell lymphoma treated with HDMTX as CNS prophylaxis reported incidence rates for any grade renal toxicity of 5‒18% (Wilson et al. 2020, 2022). (**Statement #1.3**) In addition to renal toxicity, DME increases the risk of infections and hepatic, neurological, hematological, dermatological, mucosal, pulmonary and gastrointestinal toxicities (Wilson et al. 2020, 2022; Medrano et al. 2021; Hamed et al. 2022). Mucosal and hematological toxicities are more common with infusion schedules that deliver MTX over longer periods of time at lower doses, while renal and liver toxicity occur more frequently with shorter infusions of higher MTX doses. (**Statement #1.4**)

Plasma concentration profiles of MTX following infusion of HDMTX show wide variations between individuals and between treatment cycles in individual patients, even when using the same dose and duration of infusion, and it is not possible to predict reliably which patients will develop DME (Barreto et al. 2022). Serial measurement of serum MTX concentrations following HDMTX administration is therefore routinely required to detect DME and to guide appropriate remedial interventions. Various definitions of DME are in use that vary in terms of the details of which MTX concentration thresholds and at what timepoints are considered (Alsdorf et al. 2021; Santucci et al. 2010b; Jian et al. 2023). However, serum MTX levels ≥ 10 µmol/L at 24 h (for short MTX infusions), ≥ 1 µmol/L at 42 or 48 h, or ≥ 0.3 µmol/L at 72 h after the end of infusion, are typically indicative of the presence of DME (which may or may not be associated with AKI) (Relling et al. 1994; Crom et al. 1992). (**Statement #1.5**) MTX ≥ 1 µmol/L at 42 h has been reported to occur in 22% of HDMTX cycles in pediatric patients with ALL (Relling et al. 1994).

Relevant increases in creatinine levels within 24–36 h of initiation of HDMTX may provide an early indication of DME. (**Statement #1.6**) The predictive value of plasma creatinine levels within the first 24–36 h of HDMTX initiation has been demonstrated in studies of pediatric ALL. In separate studies, 24-h serum creatinine concentrations ≥ 35.0 µM (Yang et al. 2015), a 50% increase in serum creatinine level within 24 h of HDMTX administration (Skärby et al. 2003) and a 25 µM or 50% increase within 36 h of HDMTX initiation have been found to predict DME (Schmidt et al. 2019). Reductions in urine output, fluid balance gain and weight increase following HDMTX initiation may also indicate AKI and help to predict DME (Howard et al. 2016).

### Risk factors for delayed methotrexate elimination

Evaluating the risk for development of DME and the balance of benefit to risk with HDMTX for each patient requires clinical judgement based on a holistic assessment of patient and disease characteristics. Risk factors for DME are presented in Table 2. Presence of renal impairment prior to the initiation of HDMTX is a leading risk factor for DME. MTX is predominantly cleared by the kidneys, with more than 90% eliminated unchanged in the urine (Widemann and Adamson 2006). (**Statement #2.1**) Consequently, patients with renal impairment are at increased risk of DME after HDMTX treatment (Nakano et al. 2021; Sun et al. 2022; Yang et al. 2018; Misaka et al. 2020).

**Table 2:** Factors associated with an increased risk of delayed methotrexate elimination

Factors associated with an increased risk of delayed methotrexate elimination (Howard et al. 2016; Nakano et al. 2021; Jian et al. 2023; Sun et al. 2022; Yang et al. 2018; Misaka et al. 2020; Orgel et al. 2021; Wang et al. 2020b)
• Renal impairment prior to administration of HDMTX
• Excess body weight (BMI ≥ 25 kg/m^2^)
• Frailty
• Third spacing – pleural effusion or ascites
• Fever and/or infection
• Tumor lysis
• Diabetes or hypoalbuminemia
• Drug interactions (see Table 3)

BMI, body mass index; HDMTX, high-dose methotrexate

Other clinical risk factors for DME include frailty, excess body weight, presence of pleural effusion or ascites, sepsis, fever/infection, tumor lysis, diabetes or hypoalbuminemia, Down syndrome, and concomitant use of drugs that are nephrotoxic or interfere with MTX elimination (Howard et al. 2016; Thachil 2007; Jian et al. 2023; Orgel et al. 2021; Wang et al. 2020b). (**Statement #2.2**) Renal impairment should be considered alongside these factors and particularly serum MTX levels when assessing the risk of MTX toxicity and the risk: benefit of proceeding with HDMTX. Renal dysfunction of any grade, including mild impairment (e.g. creatinine clearance < 60 mL/min), may increase the risk of toxicity during HDMTX treatment. (**Statement #2.3**)

Older patients are at greater risk of developing DME (Schwartz et al. 2006). However, it has been reported that HDMTX is feasible for the majority of older (≥ 60 years) patients (Martinez-Calle et al. 2022). The decision as to whether to initiate HDMTX in older patients should take into consideration the general fitness of each patient as well as the profile of risk factors present; most importantly renal function. (**Statement #2.4**)

Given that MTX is eliminated predominantly via the kidneys, drugs that have nephrotoxic effects or reduce renal excretion may potentially increase the risk of DME. Potential drug interactions with MTX are summarized in Table 3. In particular, acetylsalicylic acid and non-steroidal anti-inflammatory drugs as well as certain antibiotics (including penicillin and sulfonamides) interfere with MTX elimination and should be avoided in patients receiving HDMTX (medac GmbH 2022). It appears reasonable to use non-nephrotoxic antibiotic compounds for which interference with MTX elimination has not been reported (e.g., carbapenems). As a weak acid, MTX is extensively bound to albumin and can be displaced by other acidic drugs (medac GmbH 2022). Other drugs that may theoretically delay MTX elimination and require caution when considering possible HDMTX treatment include tyrosine kinase inhibitors such as imatinib and dasatinib (Pommert et al. 2021; Ramsey et al. 2019; van der Sluis et al. 2023) and proton pump inhibitors (Wang et al. 2020a). Although firm evidence about the concurrent use of these drugs is lacking, restrictive use of any comedication should always be considered in patients with delayed MTX elimination after HDMTX. Use of MTX alongside iodinated contrast agents increases the risk of renal toxicity and computed tomography and other imaging requiring contrast media should not be performed during HDMTX treatment (Schultz and Lynch 2019; Harned and Mascarenhas 2007). Consumption of acidic beverages such as colas, other carbonated drinks and fruit juices (Santucci et al. 2010a) as well as use of loop diuretics (Rastogi et al. 1985) may result in acidification of the urine, increasing the tendency for MTX to crystallize in the renal tubules and so potentially the risk of AKI and DME. Thus, consideration should be given to the potential for drug and food interactions when administering HDMTX. (**Statement #2.5**)

**Table 3:** Potential clinically-relevant drug interactions with methotrexate

Potential clinically-relevant drug interactions with methotrexate (medac GmbH 2022; Pommert et al. 2021; Ramsey et al. 2019; Wang et al. 2020a; Schultz and Lynch 2019; Harned and Mascarenhas 2007)
• Acetylsalicyclic acid and NSAIDs
• Penicillin and sulfonamides
• Tyrosine kinase inhibitors (e.g., imatinib, dasatinib)
• Probenecid and weak organic acids (e.g., pyrazoles)
• Proton pump inhibitors
• Radiographic contrast agents
• Other nephrotoxic drugs

NSAID, non-steroidal anti-inflammatory drug

Implementation of dose-reductions of HDMTX, informed by the presence of risk factors, varies across indications and between protocols. Clearly, treatment is aimed at achieving an appropriate balance of benefit to risk, and dose reductions may carry the cost of impaired efficacy. For example, studies in primary CNS lymphoma suggest that anti-tumor efficacy is likely to be significantly impaired by dose reduction in older patients and that treatment should be aimed at achieving the maximal tolerated dose (Martinez-Calle et al. 2020; Schorb et al. 2020). The decision to use a reduced dose of HDMTX should be based on a holistic assessment of anti-tumor efficacy and of risk factors and disease characteristics, paying particular attention to the presence and severity of renal impairment. (**Statement #2.6**)

HDMTX is dosed according to body surface area, calculated from the patient’s body weight and height. Generally, actual body weight can be used for this calculation but, for severely obese patients, this can result in excessively high MTX doses. Consequently, for patients with severe obesity (i.e. body mass index ≥ 40 kg/m^2^), consideration should be given to capping the dose of HDMTX, with the dose calculated according to the ideal body weight. (**Statement #2.7**)

Further research is needed to predict which patients are more likely to develop toxicity during HDMTX treatment so as better to identify patients who are suitable for treatment and guide interventions aimed at limiting toxicity, including the early use of glucarpidase. (**Statement #2.8**) Studies have found that polymorphisms in genes such as *MTHFR*, encoding proteins involved in MTX metabolism, and particularly the *SLCO1B1* gene, may contribute to DME and/or predict the risk of toxicity with HDMTX (Yang et al. 2022; Song et al. 2021). However, these potential associations require further characterization and the infrastructure required for routine testing for polymorphisms is not currently in place in most clinical centers. Consequently, testing for polymorphisms does not currently represent a practical tool for most clinical centers.

### Supportive care

Careful implementation of robust supportive measures is critical to minimizing the risk of DME-induced toxicities with HDMTX (Table 4). MTX and its metabolites are poorly soluble at acidic pH (Pitman et al. 1975). Supportive care must therefore include measures to alkalinize the urine and maintain adequate urinary flow, and thus prevent MTX crystallization in the renal tubules. To this end, hyperhydration with dextrose/saline at a flow rate of ≥ 2.5 L/m^2^/24 h should be started several hours before the administration of HDMTX and continued until achievement of non-toxic MTX levels. (**Statement #3.1**) The hydration fluid should be supplemented with sodium bicarbonate, with the concentration adjusted to achieve a urine pH of ≥ 7. (**Statement #3.2**) HDMTX should not be infused until the urine pH is ≥ 7. (**Statement #3.3**) Infections, fever and vomiting are associated with dehydration and so have the potential to increase DME-induced nephrotoxicity. Therefore, HDMTX should not generally be initiated if any of these are present. (**Statement #3.4**)

**Table 4:** Supportive measures for patients receiving high-dose methotrexate treatment

Supportive measures for patients receiving high-dose methotrexate
*Pre-HDMTX administration*
• Consider temporarily discontinuing medicines that may impair renal function or delay MTX elimination (see Table 3)
• Initiate hyperhydration (≥ 2.5 L/m^2^/24 h) and urine alkalinization several hours before the administration of HDMTX and continue until MTX clearance to non-toxic levels
• Ensure urine pH is ≥ 7 before administering HDMTX
*During HDMTX administration*
• Maintain urine output at > 100 ml/m^2^/h and urine pH ≥ 7 • Avoid weight gain
*After HDMTX administration*
• Continue hyperhydration and urine alkalinization
• Administer leucovorin rescue
• Monitor closely for DME and promptly implement remedial measures, including glucarpidase, when appropriate

DME, delayed methotrexate elimination; HDMTX, high-dose methotrexate; MTX, methotrexate

Hyperhydration carries the potential risk of fluid overload and the associated risks of pleural effusion, pulmonary edema and exacerbation of congestive heart failure (Howard et al. 2016). In patients with rapid weight gain or other signs of fluid retention, loop diuretics should be used to maintain diuresis and avoid fluid overload. Loop diuretics may also be considered to maintain urinary flow in selected patients with severe renal impairment. Acetazolamide can maintain diuresis but, unlike loop diuretics, does not acidify the urine and so may be considered for patients with inadequate urine alkalinization (Shamash et al. 1991). (**Statement #3.5**)

Administration of HDMTX is routinely accompanied by leucovorin (folinic acid) rescue to reduce the risk of toxicity. Whereas MTX primarily inhibits synthesis of folate by dihydrofolate reductase, leucovorin provides an alternative supply for synthesis and thus rescues the toxic effect of MTX. Various protocols for the use of leucovorin rescue are available and protocols for HDMTX administration often include guidance on leucovorin rescue. Rescue is typically started 24–36 h after the start of the MTX infusion with leucovorin, then given every 6 h at a dose adjusted according to the serum MTX concentration. Leucovorin should not be initiated earlier than 24 h after the start of MTX infusion to avoid potentially neutralizing the anti-tumor effects of MTX. Leucovorin rescue should continue until non-toxic levels of MTX are achieved (Howard et al. 2016). (**Statement #3.6**)

### Monitoring

Regular serial measurement of serum MTX and creatinine levels following initiation of HDMTX is essential for detecting DME and allowing timely intervention to avoid DME-induced toxicity. Although most clinics will rely on immunoassay testing to assess serum MTX concentrations, it should be recognized that the immunoassays do not reliably distinguish between MTX and its inactive metabolites, glutamate and 2,4-diamino-N-10-methylpteroic acid (DAMPA). Treatment with glucarpidase acts to reduce DME-induced toxicity by cleavage of MTX into DAMPA and, consequently, immunoassays overestimate the level of active MTX following administration of glucarpidase (Descoeur et al. 2022). The labelling for glucarpidase notes that DAMPA interference can occur in the 48 h after glucarpidase administration (SERB SAS 2024) but recent evidence suggests that discrepancies can persist for significantly longer than previously recognized (Kibby and Trinkman 2024). If available, high performance liquid chromatography-based assays provide a more reliable measure of MTX levels, particularly in the first few days following glucarpidase treatment. (**Statement #4.1**)

Serum MTX levels should be determined at regular intervals starting from 24 h after infusion of HDMTX (e.g. 24, 42, 48 and 72 h), with testing repeated at least every 24 h until discharge criteria are met (e.g. serum MTX concentration < 0.1 µmol/L). (**Statement #4.2**)

Renal function should also be monitored regularly, with creatinine, glomerular filtration rate or both determined at least every 24 h, starting 24 h after the initiation of HDMTX treatment. Closer monitoring of renal function, including cystatin C where available (Lees et al. 2024), is warranted if DME is suspected. (**Statement #4.3**) Other regular assessments should include clinical signs, fluid balance, weight, urine output and urine pH. (**Statement #4.4**)

Practice regarding discharge of patients varies between centers. In some hospitals, patients remain as in-patients under close supervision until the serum MTX level is < 0.1 µmol/L, renal function and electrolytes are stable, and the patient is clinically well with no significant fluid overload. Other centers consider discharging patients on Day 3 after HDMTX infusion if MTX kinetics at 48 h are favorable and creatinine stable. (**Statement #4.5**)

### Use of glucarpidase

Glucarpidase may be indicated to reduce toxic plasma MTX concentrations in adults and children (aged 28 days and older) with DME or at risk of MTX toxicity (SERB SAS 2024). Identification of patients who may benefit from administration of glucarpidase should be based on plasma MTX levels, taking into account factors including renal function, clinical signs and/or risk of MTX toxicity (**Statement #5.1**) The decision relies on clinical judgement but glucarpidase may be considered when plasma MTX concentrations are two standard deviations above the mean expected MTX plasma concentration based on the time and dose of MTX administered, especially if renal function is impaired. An online tool is available at https://mtxpk.org/ that uses a pharmacokinetic model to determine the concentration vs. time curve for each patient and overlay the results on the population-predicted curve for the MTX dose (Taylor et al. 2020). Glucarpidase use may also be considered based on plasma MTX concentrations exceeding thresholds of 50 µmol/L at 24 h, 30 µmol/L at 36 h, 10 µmol/L at 42 h or 5 µmol/L at 48 h after starting MTX infusion (SERB SAS 2024). (**Statement #5.2**)

According to label, glucarpidase should be administered as a single dose of 50 units/kg by bolus intravenous injection over 5 min in patients with established DME or at risk of MTX toxicity (SERB SAS 2024). Glucarpidase is supplied as a lyophilized powder in vials of 1,000 units that must be reconstituted in 1 ml of sterile 0.9% sodium chloride solution before injection. (**Statement #5.3**)

Once DME has been diagnosed and the need for glucarpidase use determined, glucarpidase should be given within 60 h, and ideally within 48 h, of the start of MTX infusion, as later administration may not be effective in preventing DME-induced toxicities (SERB SAS 2024). (**Statement #5.4**) If stocks of glucarpidase are not maintained locally, arrangements should be in place that allow access to sufficient supplies of glucarpidase within 24 h, and ideally in less than 12 h. (**Statement #5.5**)

Glucarpidase rapidly metabolizes circulating MTX but does not act on intracellular MTX. Consequently, there is a risk of rebound MTX toxicity due to release of MTX released from intracellular and extracellular tissue spaces after the activity of glucarpidase in plasma starts to fall (from ~ 48 h after dosing). The rebound level of MTX is generally substantially lower than that prior to the administration of glucarpidase (Widemann et al. 2014) and may not be clinically relevant. While MTX levels after administration of glucarpidase can be monitored with an HPLC-based assay and can detect MTX rebound, this is not usually necessary. (**Statement #5.6**)

Leucovorin is a substrate for glucarpidase and so co-administration of leucovorin may interfere with the activity of glucarpidase (Ramsey et al. 2018). Therefore, leucovorin should be stopped at least 2 h prior to and restarted only at least 2 h after glucarpidase infusion. Leucovorin should then be continued until serum MTX levels are undetectable. (**Statement #5.7**)

### Other strategies for treating delayed methotrexate elimination

In patients with DME, hydration can be intensified and leucovorin dose increased alongside administration of glucarpidase, with dosing according to relevant treatment protocols (Cerminara et al. 2019). In addition to its role in supportive care, high doses of leucovorin may also be an alternative treatment for DME when glucarpidase is unavailable or unsuitable (Flombaum et al. 2018) (**Statement #6.1**), although some data suggest this may reduce the efficacy of MTX (Skärby et al. 2006).

High-flux hemodialysis (HFHD) can be effective in clearing circulating MTX. However, the technique may be associated with a rebound of MTX levels post-dialysis, potentially to levels even higher than those pre-procedure (Widemann et al. 1997). The technique is also laborious and time-consuming and carries the risk of infection and bleeding associated with vascular access. However, HFHD may provide an effective alternative when glucarpidase is not available and for selected patients (such as those with severe renal impairment, disruption of electrolyte homeostasis and oligoanuria) (Kitchlu and Shirali 2019). HFHD should only be attempted following consultation with a nephrologist with experience in this technique (Ghannoum et al. 2022). (**Statement #6.2**)

There is insufficient evidence to support the use of activated charcoal or binding agents such as cholestyramine in the treatment of toxicity due to delayed MTX elimination. **(Statement #6.3**)

## Discussion

The consensus statements described in this manuscript represent our collective opinion based on clinical experience and available evidence. Strengths of our consensus include that we as a group bring together experience across the range of oncological indications for HDMTX encompassing both children and adults. Limitations include the relatively limited evidence base of robust clinical studies on which to base recommendations, such that personal experience and expert opinion is important. Emerging evidence of glucarpidase’s positive impact on clinical outcomes includes data from a controlled observational clinical study involving 684 adults with HDMTX-associated AKI treated in US cancer centers, demonstrating that receipt of glucarpidase to be associated with 2.43-fold higher adjusted odds of renal recovery (95% confidence interval, 1.38–4.27) compared to control patients without glucarpidase treatment (Gupta et al. 2023). Of note, a greater benefit was evident when glucarpidase was administered within 60 h of starting HDMTX. Receipt of glucarpidase was also associated with increased likelihood of recovery from neutropenia and normalization of liver enzymes. Retreatment with HDMTX after glucarpidase appears to be feasible (Christensen et al. 2012). However, full recovery from complications caused by previous HDMTX treatment, and avoidance of potential precipitating conditions, appear to be reasonable steps before readministration of HDMTX is considered. The most frequently reported adverse reactions to glucarpidase include paresthesia (2%) and flushing (2%) (European Medicines Agency 2022), but as with any intravenously administered protein, healthcare providers should be aware of its immunogenic potential. While cost implications may influence use, economic modelling based on the US setting suggests that consistent, timely intervention with glucarpidase would be associated with improved clinical outcomes and shorter duration of hospitalization versus current clinical practice. The same modelling reported that such use of glucarpidase would also be associated with cost savings when compared to delayed glucarpidase treatment or hemodialysis (Kala et al. 2023).

With respect to the limited clinical evidence on which to base clinical decisions, various protocols for HDMTX and glucarpidase treatment have been in use according to indication and across different centers. These include their own guidance for glucarpidase/supportive treatment. For example, the ALLTogether protocol (Heldrup and Schmiegelow 2023) describes an optional guideline, while mentioning national guidelines as well as the recently available free web-based clinical decision support tool, https://mtxpk.org/, which has been designed to guide physicians when administering glucarpidase to manage DME (Taylor et al. 2020). Given there is insufficient evidence to favor one protocol over another, the consensus statements are somewhat broad to reflect this diversity in practice. The aim of the consensus process was to develop a concise set of recommendations that can be usefully implemented and inform clinical practice.

Treatment incorporating HDMTX infusions continues to be an important option for treatment of a range of adult and childhood cancers. When used with appropriate supportive measures, including hyperhydration, urine alkalization and leucovorin rescue, and with careful attention to risk factors for DME and MTX toxicity, use of HDMTX can offer a positive benefit: risk profile for most patients, including the elderly. Early recognition of DME is critical in allowing timely intervention including glucarpidase, intensified hydration, and high-dose leucovorin in order to rapidly reduce MTX levels and prevent serious toxicity.

## Data Availability

Further details of the collection of data in the consensus process are available from the authors upon reasonable request.
